# Influence of *Eimeria maxima* coccidia infection on gut microbiome diversity and composition of the jejunum and cecum of indigenous chicken

**DOI:** 10.3389/fimmu.2022.994224

**Published:** 2022-09-05

**Authors:** Endashaw Jebessa, Lijin Guo, Xiaolan Chen, Semiu Folaniyi Bello, Bolin Cai, Mekonnen Girma, Olivier Hanotte, Qinghua Nie

**Affiliations:** ^1^ Department of Animal Genetics, Breeding and Reproduction, College of Animal Science, South China Agricultural University, Guangzhou, China; ^2^ Guangdong Provincial Key Lab of Agro-Animal Genomics and Molecular Breeding and Key Lab of Chicken Genetics, Breeding and Reproduction, Ministry of Agriculture, Guangzhou, China; ^3^ LiveGene – Centre for Tropical Livestock Genetics and Health (CTLGH), International Livestock Research Institute (ILRI), Addis Ababa, Ethiopia; ^4^ School of Life Sciences, Chongqing University, Chongqing, China; ^5^ School of Life Sciences, University of Nottingham, University Park, Nottingham, United Kingdom

**Keywords:** *Eimeria maxima*, infection, gut microbiome, 16S rRNA, chicken

## Abstract

Coccidiosis is an economically significant protozoan disease and an intracellular parasite that significantly impacts poultry production. The gastrointestinal tract microbiota plays a central role in host health and metabolism, and these microbes enhance chickens’ immune systems and nutrient absorption. In this study, we analyzed the abundance and diversity of microbiota of the jejunum and cecum of a dual-purpose indigenous Horro chicken following *Eimeria maxima* infection. We compared microbial abundance, composition, and diversity at the 4- and 7- days post-infection using 16S rRNA gene sequencing. We obtained, on average, 147,742 and 132,986 high-quality sequences per sample for jejunum and cecum content, respectively. Firmicutes, Proteobacteria, Campilobacterota and Bacteroidota were the major microbial phylum detected in the jejunum content. Firmicutes were the dominant phylum for 4- and 7-days jejunum control groups accounting for (>60% of the sequences). In the infected group Campilobacterota was the dominant phylum in the jejunum (> 24% of sequences) at 4-and 7-days post-infection groups, while Proteobacteria was predominant at 4- and 7-days post-infection of the cecum (> 40% of the sequences). The microbial genus *Lactobacillus* and *Helicobacter* were found in the jejunum, while *Alistipes*, *Barnesiella* and *Faecalibacterium* were detected in the cecum. In the jejunum, *Helicobacter* was dominant at 4 -and-7 days post-infection (≥24%), and *Lactobacillus* was dominant at 4 -and 7- days in the control group (> 50%). In 4- and 7-days post-infection, *Alistipes* genus was the more prevalent (> 38%) in the cecum. Thus, clear differences were observed in the bacterial microbiota distribution and abundance between the jejunum and cecum, as well as between infected and control groups for both tissues. The results indicate that chicken intestinal microbial imbalance (dysbiosis) is associated with *Eimeria* parasite infection and will likely affect the host-microbial non-pathogenic and pathogenic molecular interactions.

## Introduction

Protozoan parasites of the genus *Eimeria* are responsible for *coccidiosis*, an intracellular parasite that has a major impact on the poultry sector. As reported in a recent study, *Eimeria* species can cause damage to the intestinal parts and pose a significant risk to global poultry production (1). *Coccidiosis* causes a major economic loss in the world poultry industry, as of the reduction in production efficiencies, such as low growth rate, high mortality, decreased egg production and nutrient malabsorption (2). The *Eimeria coccidiosis* infection process is rapid and parasite replication in host cells with extensive damage to the chicken intestinal mucosa. Coccidiosis directly impacts animal health and welfare through its influence on the enteric microbiota and its effects on chicken health and production (3). *Eimeria maxima* are one of the most prevalent causes of coccidiosis (4). However, poultry is affected by seven different species of *Eimeria* (*E. tenella, E. necatrix, E. acervulina, E. maxima, E. brunetti, E. mivati, E. mitis, E. hagani and E. praecox*) that invade the gastrointestinal tract causing considerable damage to the epithelial layer (5). *Eimeria maxima* infection commences with the invasion of the jejunal epithelial cells of the chicken (6) leading to lower expression of digestive enzymes and nutrient transporters (7). *Eimeria coccidia* infection may have, a profound impact on the nutritional landscape of the chicken gastrointestinal tract (8). The intestinal damage caused by *Eimeria* parasit*e* colonization not only affects epithelial cells of chicken, but it causes high disruption of intestinal tract microbial communities, then promotes colonization and proliferation of chicken pathogens, therefore, increasing chicken mortality (9–11).

Microbiota is a collective terminology for all microorganisms, including bacteria, yeasts, filamentous fungi, and viruses, that live within all animals and provide crucial signals for the development and function of the immune system (12). The microbiota can promote resistance to colonization by pathogenic species (13). Large-scale human studies further demonstrated that the gut microbiome is mainly shaped by environmental features (14). The intestinal microflora, which exhibits high diversity, is maintained in a relative balance critical to the host’s health (15). Chickens’ gastrointestinal tracts are ports of diverse and complex microbiota that play a protective barrier by attaching to the epithelial walls of the enterocyte (16). Chicken intestinal microbiota includes diverse bacterial species (17), and different microbial communities are found in various sections of the chicken intestinal tract, with the most dominant phyla being Firmicutes, Bacteroides, and Proteobacteria (18–20). The factor affecting the intestinal microbiota composition are age, sex, breed, diet, and pathogens (21). Feed’s physical form and chemical composition affect digestibility and nutrient absorption, which influence the chicken to gut microbial composition (22).

*Eimeria* species infections disrupt gastrointestinal tract homeostasis and alter the operational taxonomic units (OTUs) composition of the chicken intestinal bacterial microbiota (23). For example, the genus *Lactobacillus* is decreased in abundance following *Eimeria* parasite infection, contributing to gastrointestinal tract damage (24). *Eimeria maxima* coccidiosis infection affects the diversity and function of the microbiota of the chicken gastrointestinal tract, resulting in serious consequences to the overall health and productivity. Even though there is significant damage to the chicken gastrointestinal tract by *Eimeria maxima*, little is known about its influence on the overall enteric microflora diversity and functions.

The study was carried out on non-infected control and infected chicken intestinal bacterial microbiota with *Eimeria maxima*, investigating the microbial composition and abundance of the jejunum and cecum contents. 16S rRNA metagenomic sequences were used to analyze the diversity of jejunal and cecal bacterial microbiota comprehensively. Our purpose was to understand the early infection’s impact on *Eimeria maxima* on the microbial diversity and its function for an indigenous dual-purpose chicken breed (Horro).

## Materials and methods

### Animal ethics statement

All the experimental procedures in this study were conducted in compliance with animal welfare protocols and made all efforts to minimize animal suffering following relevant guidelines and regulations of the Institute Animal Care the Use Committee (IACUC) of ILRI (Nairobi, Kenya) (IACUC-RC2019-01).

### Experimental design and sample collection

This study used fertilized eggs of the Ethiopian Horro chicken breed. Eggs were incubated in an automatic incubator at 37.5°C with 78% relative humidity. A total of 48 one-day-old Horro chickens were randomly divided into two groups (n = 24), the infected group (IG) and the control group (CG), with four replicates per group (n = 6); they were kept in the starter brooder units (ILRI, Addis Ababa, Ethiopia). All chickens were maintained in a temperature-controlled environment based on a standard protocol and provided with feed and water *ad-libitum*. For the *E. maxima* challenged group, chickens were infected by oral gavage on day 21 with 2 ml containing 7x10^4^ sporulated oocysts/bird. To characterize the impact of *Eimeria maxima* on the gastrointestinal tract of Ethiopian Horro chicken breeds, 32 chickens were randomly selected from the infected and control groups and humanely euthanized at 24 and 28 days (8 chickens/group for each respective day). The entire gastrointestinal tract (GIT) of each individual selected bird was dissected and 200 mg of the jejunum and cecum contents were collected into a 2 ml sterile plastic tube. Then, the collected tubes were stored at –80°C until microbial DNA extraction.

### DNA extraction and preparation

Total microbial DNAs of chickens’ jejunum and cecum content on days 4 -and 7 post-infections were isolated from three individuals, each from the infection and control group, using the QIAamp DNA Stool Mini Kit (Qiagen, Hilden, Germany), according to the manufacturer’s instructions and stored at −80°C. Before library construction, all samples were briefly tested for DNA degradation and potential contamination on a 1.5% agarose gel electrophoresis. The DNA purity was tested using a 2000 NanoDrop, (ThermoFisher, Scientific, USA), with all samples within the expected OD260/280 = 1.8 – 2.0 ratio, supporting optimal DNA purity.

### PCR amplification

Bacterial DNA amplification was carried out by targeting the V3–V4 region of the 16S rRNA gene using a specific primer with a barcode. The V3–V4 region was amplified using the conserved PCR primers: 341F (5’ CCTAYGGGRBGCASCAG -3’) and 806R (5’ GGACTACNNGGGTATCTAAT -3’). All PCR reactions were carried out with Phusion^®^ High-Fidelity PCR Master Mix (New England Biolabs). PCR products were run on a 2% agarose gel for quantification. Samples with a bright strip band between 400 - 450 bp were chosen for sequencing.

### Library preparation and Illumina sequencing

The sequence libraries were generated using the NEBNext Ultra DNA Library Pre^®^Kit for Illumina, following the manufacturer’s instruction, and index codes were added. For size distribution detection, the library quality was assessed on the Qubit@ 2.0 Fluorometer (Thermo Scientific) and Agilent Bioanalyzer 2100 system. The effective library concentration and data yield required were sequenced on an Illumina NovaSeq platform and 250 bp paired-end reads were generated.

### Bioinformatic data analysis

Quality filtering on the raw tags was performed under specific filtering conditions to obtain high-quality clean tags (25) according to the QIIME (V1.7.0) (26) (http://qiime.org/scripts/split_libraries_fastq.html) quality-controlled process. OTU cluster and taxonomic annotation sequence analyses were performed by UPARSE software (27) (http://drive5.com/uparse/), using all the effective tags. Sequences with ≥ 97% similarity were assigned to the same OTUs. For each representative sequence, the mothur method was used to annotate the SSU rRNA database of SILVA138 Database (http://www.arb-silva.de/) (28), for species annotation at each taxonomic rank (threshold:0.8~1) (29). To compare multiple sequences rapidly, phylogenetic diversity and relationship of all OTUs representative sequences were performed using the MUSCLE software (30). OTUs abundance information was normalized using a standard sequence number corresponding to the sample with the least sequences. Subsequent alpha and beta diversity analyses were performed based on this output normalized data. All calculations were performed with QIIME (VERSION 1.7.0) and displayed with the R software (Version 2.15.3).

### Statistical analysis

Species diversity was assessed with the use of Shannon, Simpson, Chao, and Ace indices calculated by QIIME software. The hierarchical clustering heat map of phylum and genus levels was calculated by QIIME software and displayed with R software. Principal component analysis (PCA) is a method to extract principal components and structures in data by using orthogonal transformation and reducing the dimensionalities of data (31). Principal Component Analysis (PCA) was performed to get principal coordinates and calculated by (un) weighted UniFrac distance. *P*-value was calculated following the permutation test. The difference was considered significant at *P* < 0.05, while the q-value was calculated correcting for Benjamini and Hochberg False Discovery Rate (32). Results are presented as means ± standard deviation (SD).

## Results

### Jejunum and cecum 16S rRNA sequences

After quality filtering and assembly, we obtained a total of 3,368,734 sequences, with an average sequence number of 147,742 and 132,986 sequences per sample for the jejunum and cecum, respectively (**Supplementary Table S1**). The average sequence lengths for jejunum and cecum were 420 bp and 418 bp, respectively (**Supplementary Table S1**). The operational taxonomic units (OTUs) generated at 97% sequence identity were considered for functional taxonomic units clustering and calculating the statistical indices of alpha and beta diversity. The average OTUs of the different samples were collected, such as total tags, taxon tags, unclassified tags, unique tags, and OTUs annotation data (**Supplementary Figure S1**).

### Rarefaction curve and OTUs cluster

**Figure 1:**
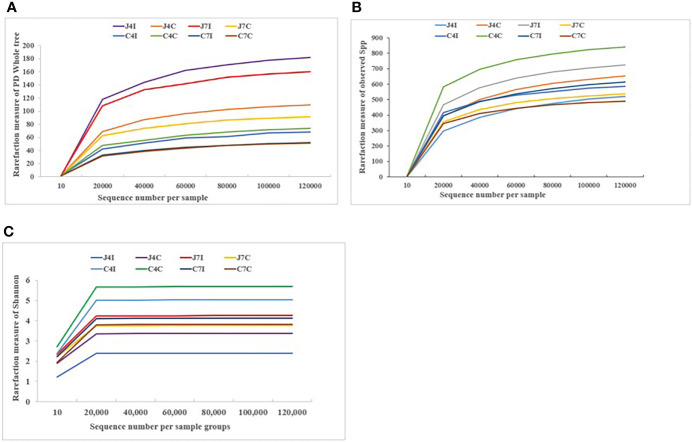
Rarefaction curve plot of sequences number per sample group. **(A)** Observed species. **(B)** PD Whole tree. **(C)** Shannon.

The rarefaction curves tend to achieve saturation, indicating that the sequencing amount of the microbiota of the eight sample groups was enough to assess the richness and microbial community diversity at the 97% similarity threshold. The reads we used for the following bacterial community diversity analysis and alpha-diversity metrics, such as observed species, Shannon, Simpson, Chao1, ACE, good coverage, and PD Whole Tree of OTUs were established for 4- and 7-days post-infection and control groups for both chicken jejunum and cecum (**Supplementary Table S2**). Rarefaction curves (**Figures 1A-C**) become flat and tend to plateau with more data showing that the number of OTUs we analyzed for each sample group was sufficient.

### Analysis of microbial composition and abundance in chicken intestine

**Figure 2:**
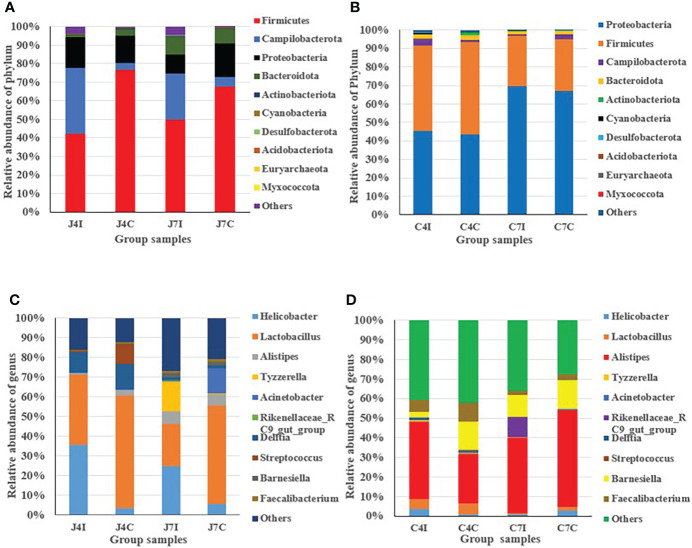
Distribution and relative abundance of the most common microbial phyla and genus in different types of samples. **(A)** Phyla - jejunum. **(B)** Phyla – cecum. **(C)** Genus - jejunum. **(D)** Genus - cecum.

From the top ten microbiome abundance at the phylum level, Firmicutes (42.33%), Campilobacterota (35.45%), Proteobacteria (16.61%) and Bacteroidota (1.06%) were the most abundant at 4 days post-infection for the jejunum while Firmicutes (76.49%), Proteobacteria (14.85%), Campilobacterota (3.64%) and Bacteroidota (3.56%) were the most abundant in the control group (**Figure 2A**). At 7 days, post-infection Firmicutes (49.33%), Campilobacterota (24.90%), Proteobacteria (10.19), and Bacteroidota (9.77%) were the most abundant in the jejunum, while for the control group it was Firmicutes (67.42%), Proteobacteria (17.93%), Bacteroidota (8.04%) and Campilobacterota (5.48%) (**Figure 2A**). Chicken cecum at 4-and 7-days post-infections was similarly analyzed for microbiome composition. The most common microbial phyla were Firmicutes and Proteobacteria at 4 days post-infection and control group (**Figure 2B**). Proteobacteria were highly dominant in the cecum at 7 days post-infection, and in the control group, accounting for > 65%, Campilobacterota and Bacteroidota were the most abundant (**Figure 2B**).

In the jejunum, at the genus level, *Lactobacillus* (35.83%), *Helicobacter* (35.45%), and *Delftia* (10.45%) were the most common genus at 4 days post-infection. In the 4 days control groups, *Lactobacillus* (56.9%) was the most common, with the other main genera being (*Delftia* (12.78%), *Streptococcus* (10.08%), *Helicobacter* (3.62%) and *Alistipes* (2.79%), present at a much lower frequency (**Figure 2C**). At 7 days post-infection, we found *Helicobacter* (24.9%), *Lactobacillus* (21.53%), *Tyzzerella* (14.88%), and *Alistipes* (6.25%), while in the control groups the most common genus was *Lactobacillus* (50.14%), Acinetobacter (12.90%), *Alistipes* (5.68%) and *Helicobacter* (5.48%) (**Figure 2C**).

In the cecum, at 4 days post-infection, the most common genus was *Alistipes* (39.58%), *Faecalibacterium* (5.89%), *Lactobacillus* (5.06%), *Helicobacter* (3.60%), and *Barnesiella* (3.11%), and for the control group genus *Alistipes* (25.04%), *Barnesiella* (14.43%), *Faecalibacterium* (9.87%) and *Lactobacillus* (5.65%) (**Figure 2D**). At 7 days post-infection we found *Alistipes* (38.33%), *Barnesiella* (10.84%), *Rikenellaceae_RC9_gut_group* (10.38%) and *Faecalibacterium* (2.44%). *Alistipes* (49.21%) were highly abundant at 7 days in the control group, with the other genus *Barnesiella* (14.45%), *Helicobacter* (2.99%), and *Faecalibacterium* (2.75%) also common (**Figure 2D**). Overall, the results indicated that in the chicken intestinal tract microbial communities varied in their compositions in controlled as well as in infected groups for both the jejunum and the cecum.

### Alpha diversity analyses of the microbial community

**Figure 3:**
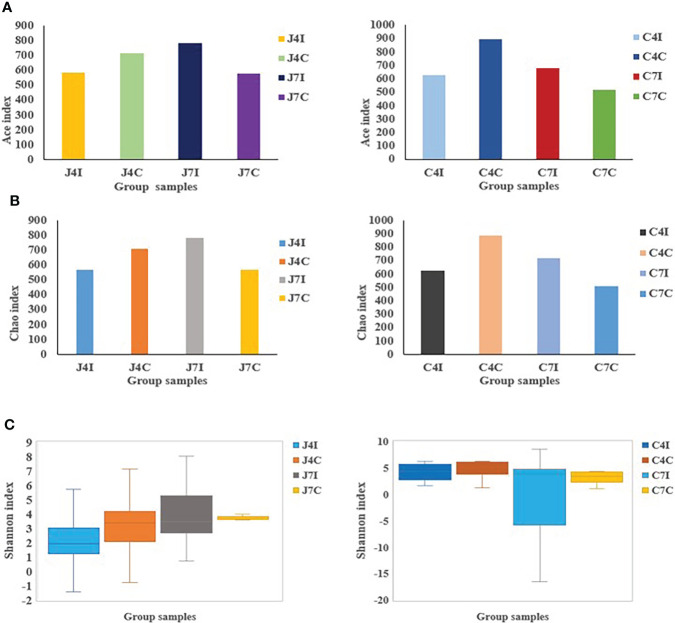
OTUs Alpha diversity analysis: **(A)** Ace index of jejunum and cecum. **(B)** Chao index of jejunum and cecum. **(C)** Shannon index analysis of jejunum and cecum.

Alpha diversity represents species richness, evenness, and diversity. Chao is a non-parametric method for estimating the number of species in an intestinal microbial community (**Figure 3A**). At the same time, the Ace index represents the community richness of the gut microbiota (**Figure 3B**). The greater the Chao and Ace index, the higher the expected species richness of the microbiota. The Shannon index measures community diversity, which estimates the number of microbial species present, and the higher value of the Shannon index, the higher the diversity (**Figure 3C**). No significant (*P* > 0.05) differences were observed between the Ace, Chao, and Shannon indexes.

### Shared and unique microbial populations

**Figure 4:**
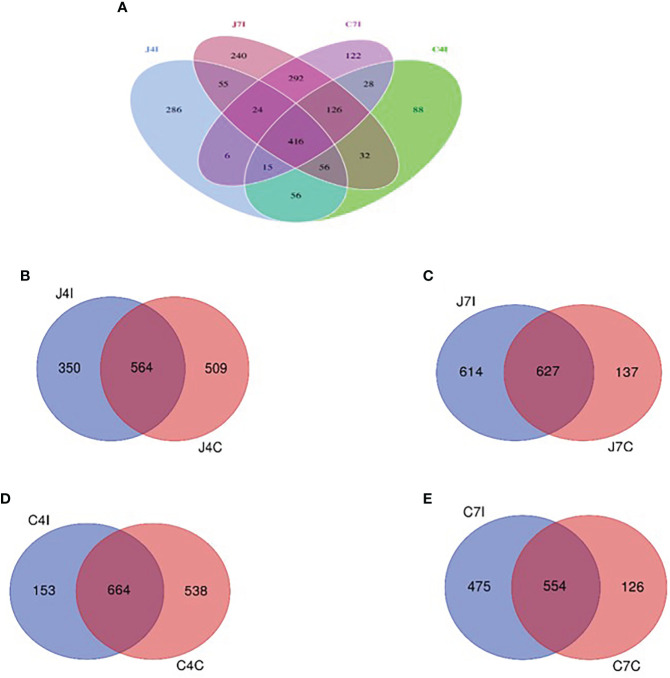
Venn diagram showing the number of unique and shared OTUs in jejunum and cecum content microbial. **(A)** The jejunum and cecum OTUs at fourth- and seventh-day post-infection. **(B)** OTUs of jejunum at fourth-day post-infection and control group. **(C)** OTUs of jejunum at seventh-day post-infection and control group. **(D)** OTUs of cecum on the fourth-day post-infection and control group. **(E)** OTUs of cecum at seventh-day post-infection and control group.

To further study the microbial community of the chicken GIT section with *E. maxima* infection, we analyzed unique and shared OTUs of jejunum and cecum from infected and control groups. Afterwards, we identified unique and shared OTUs of infected and controlled chicken jejunum and cecum. A total of 1842 OTUs were detected, and unique taxon ranged from 88 to 286 OTUs at 4- and 7-days post-infection (jejunum and cecum) (**Figure 4A**). Fifty-five OTUs were shared between 4- and 7- days post-infection in the jejunum, and 28 OTUs for the cecum. Unique OTUs were also more common in the jejunum (days 4 and 7) compared to the cecum (**Figure 4A**).

Whereas 564 OTUs were shared at 4 days, post-infection, and control groups jejunum (J4I and J4C), 350 unique OTUs in jejunum post-infection (J4I) and 509 in jejunum control (J4C) were identified. The number of unique OTUs in the J4C was higher than in the J4I, suggesting that the microbial composition in the chicken GIT might decrease following *E. maxima* infection (**Figure 4B**). Unique OTUs were detected in the two experimental groups, 614 and 137 OTUs at 7 days, post-infection, and control groups of the jejunum (J7I and J7C), respectively. While 627 OTUs were shared between the J7I and J7C groups (**Figure 4C**). In the cecum, 664 OTUs were shared between 4 days post-infection and control groups for the cecum (C4I and C4C), whereas 153 in C4I and 538 in C4C groups were unique (**Figure 4D**). Thus, as observed in the cecum OTUs, diversity in the control group was higher at 4 days post-infection. In the cecum tract, 554 OTUs were shared with the 7 days post-infection and control groups (C7I and C7C), while 475 and 126 OTUs were unique to the C7I and C7C groups, respectively (**Figure 4E**).

### Clustering microbial community composition

**Figure 5:**
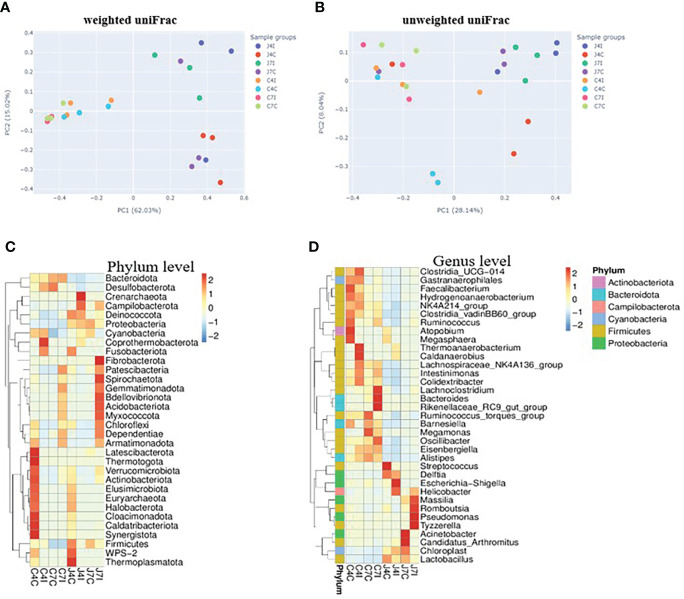
PCA analysis of operational taxonomic units from sample groups. **(A)** weighted uniFrac of jejunum and cecum groups **(B)** unweighted uniFrac jejunum and cecum groups. The different colours in the PCA plot are represents different sample groups, and the closer sample distance point has more like the microbial composition. The microbial abundance heat map shows the hierarchical distribution of the phylum and genus levels. **(C)** Microbial cluster distribution of 32 phyla. **(D)** Microbial cluster distribution of 35 genus. The colour intensity in each panel shows the percentage of microbial composition referring to the colour key value.

Weighted and unweighted uniFrac were used to investigate the beta diversity of infection and control sample groups through distance matrix PCA plotting. The similarity and difference of microbial diversity in the infection and control groups of jejunum and cecum were analyzed in a PCA plot with PC1 accounting for 62.03% and PC2 accounting for 15.02% variation in weighted uniFrac (**Figure 5A**), while for the unweighted uniFrac, the two first PC accounted for PC1, 28.14% and PC2, 8.04% of the variation (**Figure 5B**). PCA analysis clearly separates the jejunum and cecum microbiome. Each tissue sample is closer in the weighted uniFrac analysis. The more similar microbial communities among the samples, the closer the distance sample point on the PCA plot. Furthermore, clustered heat maps were performed at phylum and genus levels (**Figures 5C, D**). The microbial community heat map reveals the richness and diversity of bacterial populations in the infection and control group of jejunum and cecum samples.

### Functional analysis of jejunum and cecum microbial community

**Figure 6:**
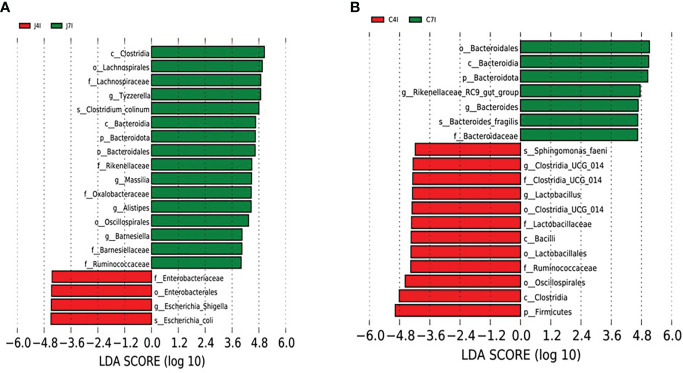
LEFSe analysis of differential OTUs along with the fourth- and seventh-day post-infection of jejunum and cecum groups. **(A)** Differential microbial enrichment of J4I and J7I **(B)** Differential microbial enrichment of C4I and C7I. The green and red horizontal colour bars represent microbial enrichment in the 4- and 7-days post-infection of jejunum and cecum, respectively.

We performed a Linear discriminant analysis Effect Size (LEfSe) functional analysis to detect the differential microbial enrichments of chicken jejunum and cecum following *Eimeria maxima* infection. The histogram of the LDA scores ((log 10) > 4)) structures exhibited a significant (*P* < 0.05) enriched microbiota difference between jejunum and cecum on different days post-infection. The LDA scores showed that the relative abundance of *Escherichia*_*Shigella*, *Escherichia_coli* and *Enterobacteriaceae* were enriched at 4 days post-infection in the jejunum. In contrast, *Tyzzerella*, *Massilia, Alistipes Barnesiella* and Bacteroidota were enriched at the 7 days post-infection in the jejunum (**Figure 6A**). Firmicutes, Ruminococcaceae, Bacteroidaceae, Oscillospirales *Lactobacillus* and *Clostridia_UCG_014* were enriched at 4 days post-infection in the cecum. On the 7 days post-infection of cecum *Bacteroides*, Bacteroidota, Bacteroidales, *Rikenellaceae_RC9_gut_group*, Ruminococcaceae and Bacteroidaceae were enriched (**Figure 6B**). Comparable enrichment analyses at 4 -and 7-days post-infection of jejunum and cecum by linear discriminant analysis (LDA) plot identify unique biomarkers bacterial species for the jejunum and cecum infection group.

## Discussion

The study analyzed Ethiopian indigenous Horro chicken jejunum and cecum microbiota composition after infection with *Eimeria maxima* to understand host-microbial interaction following infection with a pathogen. The chicken gastrointestinal tract (GIT) microbial diversity and composition are expected to play a vital role in health and growth, enhancing the immune system and nutrient absorption (33). Bacterial colonization in the gut begins immediately after the hatching, while the intestinal microbial composition is influenced by different factors such as pathological conditions, genetics, environment, age, and diet (34). Host diet can importantly impact the gastrointestinal tract microbiome, which in turn influences host metabolism and welfare (35). Microbiota diversity is expected to vary along the intestinal tract, and it undertakes change over time, following pathogen infection or changes in diet (36). It is therefore important to understand the variation of microbial composition in different sections of the chicken gastrointestinal tract. Here, we used high-throughput sequencing technology to examine host-microbial interaction following *Eimeria* coccidia infection of chicken jejunum and cecum.

A gastrointestinal tract microbial of chickens with high diversity is expected to be more stable and healthier than a low diversity one (37). The cecal microbial taxa of Firmicutes and Proteobacteria were significantly increased after the *Eimeria-tenella* infection (38). From our study, Firmicutes, Campilobacterota, Proteobacteria, and Bacteroidota were the major phyla in the chicken jejunum and cecum intestinal tract post-infection with *Eimeria maxima*. Thus, Firmicutes and Proteobacteria were highly dominant during post-infection of jejunum and cecum, respectively. In addition, Firmicutes were more abundant at 7 days post-infection than at 4 days post-infection in the jejunum. Likewise, proteobacteria were more abundant at 7 days post-infection than at 4 days post-infection in the cecum intestinal tract. So *Eimeria tenella* infection changed the abundance of the bacteria OTUs and caused cecal microbiota dysbiosis (3).

The gastrointestinal consists of important microbes that encourage the nutrition and proper development of chicken; however, the gut microbiome is affected by different *Eimeria* parasite infections, causing an imbalance in intestinal homeostasis, and driving the increment of pathogens (8). The disruption of the microbiota homeostasis has been related to pathogens infection in humans and animals (39). The host-pathogen interactions influence the host’s complex body system, including immunology, physiology, nutrition, and the gut microbiome. The major bacterial genera were the *Lactobacillus* and *Helicobacter* at the 4-and 7-days post-infection of jejunum. *Alistipes, Barnesiella and Faecalibacterium* were major genera at the 4- and 7-days post-infection of the cecum. Increasing bacterial strains abundance may increase resistance to *Eimeria* infection by inhibiting intestinal pathogens. *Lactobacillus* can modulate the inborn and developed immune system of poultry, contributing to resistance to bacterial and parasitic infection (3). Some *Lactobacillus* species will help stabilize the gut microbial composition by generating antimicrobial bacteriocins (40). In the jejunum, *Helicobacter* was the predominant genus at 7 days post-infection, while *Lactobacillus* was more dominant at 4 days post-infection. Also, *Alistipes* was the most-commonest bacterial genera on both 4 -and 7-days post-infection in the cecum. The microbial composition and diversity were different in each intestinal section (41).

Alpha diversity indexes calculations, such as Ace, Chao1, and Shannon, measure the distribution and abundance of OTUs within a specific population community (33). Microbial diversity, evenness and richness are vital indicators of the health of the gastrointestinal tract (33). The number of microbes in the gut decreases during pathogenic disruption or nutrient imbalance (42). Chao1 and Ace index values for the jejunum and cecum following *Eimeria maxima* infection on the 4 days post-infection were lower compared to their control groups. But at 7 days post-infection (jejunum and cecum) were higher in microbial richness relative to their respective control groups. Alpha diversity is strongly associated with specific gene functions, which contributes to the fundamental microbiota mechanism (43).

Gut microbial clusters were dissimilar in individual birds and for the two studied intestinal tracts. The Venn diagram showed that most of the OUTs are shared at 4 -and 7-days post-infection between the jejunum and cecum intestinal tracts. Operational taxonomic unit populations affected by *Eimeria* coccidia infection help to identify bacterial populations present in the gut, which could be useful in sustaining and restoring gut homeostasis during and after infection (44). The number of OTUs in chicken jejunum and cecum at fourth-day post-infection with *Eimeria maxima* was much less than that of control groups. On the other hand, from the 7 days, post-infection, chicken jejunum and cecum OTUs were higher in the control groups. These results illustrate that the microbiota diversity, following infection with *Eimeria maxima*, will depend on the time since the initial infection. The main effect of infection will be initially a reduction of the microbiota diversity, as illustrated by the results on day 4 post-infection, but then an increase in diversity is observed on day 7 post-infection. It illustrates the plasticity of the microbiota of the Horro chicken to respond to the infection with *Eimeria maxima*, a possible adaptive response of indigenous chicken to mitigate the detrimental effect. Comparative studies with other breeds may assess if such an adaptive mechanism is breed specific.

Principal component analysis is required to significantly reduce their dimensionality in an understandable way, such that most of the information in the data is well-preserved (45). PCA result of the microbial community in jejunum, infection and control groups were distinctly separated, while infection and control groups were closer in the cecum. Hence the intestinal microbiota composition of the jejunum sample groups was different from that of the cecum, with more separation between the control and the infected group.

Furthermore, Linear discriminate analysis (LDA) was used to further assess the difference in relative abundances of gastrointestinal microflora associated with *Eimeria maxima* infection. Thus, we calculated the LDA scores of chickens’ intestinal microflora, and we found that *Escherichia*_*Shigella*, *Escherichia*_*coli* and *Enterobacteriaceae* were significantly enriched a day 4 post-infection in the jejunum. Furthermore, nine bacterial genera were differentially expressed between days 4 -and 7 post-infections in the jejunum and cecum. The result showed that *Tyzzerella, Masala*, *Alistipes* and *Barnesiella* were over-represented at the 7 days post-infection in the jejunum, while *Escherichia_Shigella* was underrepresented at 4 days post-infection in the jejunum. In addition, cecum bacteria genera, *Bacteroides and Rikenellaceae_RC9_gut_group* were overexpressed at the 7 days post-infection, whereas *Lactobacillus* and *Clostridia_UCG_014* genera were underrepresented at the 4 days post-infection. Several studies have reported that *Lactobacillus* inhibits the growth of pathogens through the production of organic acids, therefore promoting animal growth (46).

The study revealed that *Eimeria maxima* infection influenced the gastrointestinal tract microbial community and impacted the microbiota diversity in chickens. Thus, the gut microbiome could be used to evaluate interactions between the host microbiome and parasite infection. All these results could suggest valuable information for future studies to understand how *Eimeria maxima* parasite infection influences the chicken gut microbial composition imbalance. However, it needs further research on mediating microbial communities and their role in chicken’s metabolism and health status.

## Conclusion

The current study has demonstrated the influence of *Eimeria maxima* infection on microbial diversity and its abundance in the chicken jejunum and cecum. The gastrointestinal tract microbiome composition is possibly correlated with gut pathology. It reveals the importance of accounting for microbial abundance differences while exploring the association between the *Eimeria* parasite and the chicken microbiome. The major phylum, Firmicutes, Campilobacterota, Proteobacteria, and Bacteroidota were detected in post-infection of jejunum and cecum. Chicken jejunum and cecum microbiome that difference in abundance and composition were mainly related to *Eimeria maxima* infection and may affect poultry health and production. In conclusion, *Eimeria maxima* infection of the chicken gastrointestinal tract has influenced microbial diversity, composition, and enrichment from different time points.

## Data availability statement

The data presented in the study are deposited in NCBI SRA repository as BioProject PRJNA872932.

## Ethics statement

All the experimental procedures in this study were conducted in compliance with animal welfare protocols and made all efforts to minimize animal suffering following relevant guidelines and regulations of the Institute Animal Care the Use Committee (IACUC) of ILRI (Nairobi, Kenya) (IACUC-RC2019-01).

## Author contributions

EJ carried out most of the experiment’s data analysis and drafted the manuscript. QN and OH designed the study and reviewed the manuscript. LG, XC, and BC carried out part of the experiments. SB reviewed the manuscript. MG participated in the chicken coccidia challenge experiment and sample collection. All authors read and approved the final manuscript.

## Funding

This work was supported by the Natural Scientific Foundation of China (31761143014).

## Acknowledgments

We thank ILRI, Addis Ababa poultry facility members, for cooperating with sample collection, and Foshan Standard Bio-Tech Co., Ltd for a donation of *Eimeria maxima oocysts*.

## Conflict of interest

The authors declare that the research was conducted in the absence of any commercial or financial relationships that could be construed as a potential conflict of interest.

## Publisher’s note

All claims expressed in this article are solely those of the authors and do not necessarily represent those of their affiliated organizations, or those of the publisher, the editors and the reviewers. Any product that may be evaluated in this article, or claim that may be made by its manufacturer, is not guaranteed or endorsed by the publisher.

## Supplementary material

Supplementary Table 1 Data quality control (QC) statistical analysis of samples.

Supplementary Table 2 Alpha diversity of sequence number of reads.

Supplementary Figure 1 Statistical analysis of the tags and OTUs number of each sample.

## References

[1] Attree E Sanchez-Arsuaga G Jones M Xia D Marugan-Hernandez V Blake D. Controlling the causative agents of coccidiosis in domestic chickens; an eye on the past and considerations for the future. CABI Agric Biosci (2021) 2(1):1–6. doi: 10.1186/s43170-021-00056-5 PMC8475900 34604790

[2] Lillehoj HS Kim CH Keeler CL Jr. Zhang S. Immunogenomic approaches to study host immunity to enteric pathogens. Poultry Sci (2007) 86(7):1491–500. doi: 10.1093/ps/86.7.1491 17575200

[3] Macdonald SE Nolan MJ Harman K Boulton K Hume DA Tomley FM. Effects of eimeria tenella infection on chicken caecal microbiome diversity, exploring variation associated with severity of pathology. PloS One (2017) 12(9):e0184890. doi: 10.1371/journal.pone.0184890 28934262 PMC5608234

[4] Yang X Li M Liu J Ji Y Li X Xu L. Identification of immune protective genes of eimeria maxima through cDNA expression library screening. Parasites Vectors (2017) 10(1):1–0. doi: 10.1186/s13071-017-2029-4 28209186 PMC5322808

[5] Chapman HD. Milestones in avian coccidiosis research: A review. Poultry Sci (2014) 93(3):501–11. doi: 10.3382/ps.2013-03634 24604841

[6] Huang J Liu T Li K Song X Yan R Xu L. Proteomic analysis of protein interactions between eimeria maxima sporozoites and chicken jejunal epithelial cells by shotgun LC-MS/MS. Parasites Vectors (2018) 11(1):1–0. doi: 10.1186/s13071-018-2818-4 29618377 PMC5885459

[7] Paris NE Wong EA. Expression of digestive enzymes and nutrient transporters in the intestine of eimeria maxima-infected chickens. Poultry Sci (2013) 92(5):1331–5. doi: 10.3382/ps.2012-02966 23571343

[8] Madlala T Okpeku M Adeleke MA. Understanding the interactions between eimeria infection and gut microbiota, towards the control of chicken coccidiosis: A review. Parasite (2021) 28:1–10. doi: 10.1051/parasite/2021047 34076575 PMC8171251

[9] Hauck R. Interactions between parasites and the bacterial microbiota of chickens. Avian Dis (2017) 61(4):428–36. doi: 10.1637/11675-051917-Review.1 29337611

[10] Antonissen G Eeckhaut V Van Driessche K Onrust L Haesebrouck F Ducatelle R. Microbial shifts associated with necrotic enteritis. Avian Pathol (2016) 45(3):308–12. doi: 10.1080/03079457.2016.1152625 26950294

[11] Macdonald SE van Diemen PM Martineau H Stevens MP Tomley FM Stabler RA. Impact of eimeria tenella coinfection on campylobacter jejuni colonization of the chicken. Infect Immun (2019) 87(2):e00772–18. doi: 10.1128/IAI.00772-18 PMC6346136 30510107

[12] Sekirov I Russell SL Antunes LC Finlay BB. Gut microbiota in health and disease. Physiol Rev (2010) 90:859–904. doi: 10.1152/physrev.00045.2009 20664075

[13] Sassone-Corsi M Raffatellu M. No vacancy: how beneficial microbes cooperate with immunity to provide colonization resistance to pathogens. J Immunol (2015) 194(9):4081–7. doi: 10.4049/jimmunol.1403169 PMC4402713 25888704

[14] Rothschild D Weissbrod O Barkan E Kurilshikov A Korem T Zeevi D. Environment dominates over host genetics in shaping human gut microbiota. Nature (2018) 555(7695):210–5. doi: 10.1038/nature25973 29489753

[15] Lozupone CA Stombaugh JI Gordon JI Jansson JK Knight R. Diversity, stability and resilience of the human gut microbiota. Nature (2012) 489(7415):220–30. doi: 10.1038/nature11550 PMC3577372 22972295

[16] Shang Y Kumar S Oakley B Kim WK. Chicken gut microbiota: importance and detection technology. Front Veterinary Sci (2018) 5:254. doi: 10.3389/fvets.2018.00254 PMC6206279 30406117

[17] Wei S Morrison M Yu Z. Bacterial census of poultry intestinal microbiome. Poultry Sci (2013) 92(3):671–83. doi: 10.3382/ps.2012-02822 23436518

[18] Stanley D Hughes RJ Moore RJ. Microbiota of the chicken gastrointestinal tract: influence on health, productivity and disease. Appl Microbiol Biotechnol (2014) 98(10):4301–10. doi: 10.1007/s00253-014-5646-2 24643736

[19] Gill SR Pop M DeBoy RT Eckburg PB Turnbaugh PJ Samuel BS. Metagenomic analysis of the human distal gut microbiome. Science (2006) 312(5778):1355–9. doi: 10.1126/science.1124234 PMC3027896 16741115

[20] Ley RE Hamady M Lozupone C Turnbaugh PJ Ramey RR Bircher JS. Evolution of mammals and their gut microbes. Science (2008) 320(5883):1647–51. doi: 10.1126/science.1155725 PMC2649005 18497261

[21] Kers JG Velkers FC Fischer EA Hermes GD Stegeman JA Smidt H. Host and environmental factors affecting the intestinal microbiota in chickens. Front Microbiol (2018) 9:235. doi: 10.3389/fmicb.2018.00235 29503637 PMC5820305

[22] Apajalahti J Kettunen A Graham H. Characteristics of the gastrointestinal microbial communities, with special reference to the chicken. World's Poultry Sci J (2004) 60(2):223–32. doi: 10.1079/WPS20040017

[23] Stanley D Keyburn AL Denman SE Moore RJ. Changes in the caecal microflora of chickens following clostridium perfringens challenge to induce necrotic enteritis. Veterinary Microbiol (2012) 159(1-2):155–62. doi: 10.1016/j.vetmic.2012.03.032 22487456

[24] Taubert A Behrendt JH Sühwold A Zahner H Hermosilla C. Monocyte-and macrophage-mediated immune reactions against eimeria bovis. Veterinary Parasitol (2009) 164(2-4):141–53. doi: 10.1016/j.vetpar.2009.06.003 19559532

[25] Bokulich NA Subramanian S Faith JJ Gevers D Gordon JI Knight R. Quality-filtering vastly improves diversity estimates from illumina amplicon sequencing. Nat Methods (2013) 10(1):57–9. doi: 10.1038/nmeth.2276 PMC3531572 23202435

[26] Caporaso JG Kuczynski J Stombaugh J Bittinger K Bushman FD Costello EK. QIIME allows analysis of high-throughput community sequencing data. Nat Methods (2010) 7(5):335–6. doi: 10.1038/nmeth.f.303 PMC3156573 20383131

[27] Edgar RC. UPARSE: highly accurate OTU sequences from microbial amplicon reads. Nat Methods (2013) 10(10):996–8. doi: 10.1038/nmeth.2604 23955772

[28] Wang Q Garrity GM Tiedje JM Cole JR. Naive Bayesian classifier for rapid assignment of rRNA sequences into the new bacterial taxonomy. Appl Environ Microbiol (2007) 73(16):5261–7. doi: 10.1128/AEM.00062-07 PMC1950982 17586664

[29] Quast C Pruesse E Yilmaz P Gerken J Schweer T Yarza P. The SILVA ribosomal RNA gene database project: Improved data processing and web-based tools. Nucleic Acids Res (2012) 41(D1):D590–6. doi: 10.1093/nar/gks1219 PMC3531112 23193283

[30] Edgar RC. MUSCLE: multiple sequence alignment with high accuracy and high throughput. Nucleic Acids Res (2004) 32(5):1792–7. doi: 10.1093/nar/gkh340 PMC390337 15034147

[31] Avershina E Frisli T Rudi K. *De novo* semi-alignment of 16S rRNA gene sequences for deep phylogenetic characterization of next-generation sequencing data. Microbes Environments (2013) 28(2):211–216. doi: 10.1264/jsme2.ME12157 23603801 PMC4070667

[32] White JR Nagarajan N Pop M. Statistical methods for detecting differentially abundant features in clinical metagenomic samples. PloS Comput Biol (2009) 5(4):e1000352. doi: 10.1371/journal.pcbi.1000352 19360128 PMC2661018

[33] Choi KY Lee TK Sul WJ. Metagenomic analysis of chicken gut microbiota for improving metabolism and health of chickens–a review. Asian-Australasian J Anim Sci (2015) 28(9):1217. doi: 10.5713/ajas.15.0026 PMC4554860 26323514

[34] Corrigan A de Leeuw M Penaud-Frézet S Dimova D Murphy RA. Phylogenetic and functional alterations in bacterial community compositions in broiler ceca as a result of mannan oligosaccharide supplementation. Appl Environ Microbiol (2015) 81(10):3460–70. doi: 10.1128/AEM.04194-14 PMC4407213 25769823

[35] Manor O Dai CL Kornilov SA Smith B Price ND Lovejoy JC. Health and disease markers correlate with gut microbiome composition across thousands of people. Nat Commun (2020) 11(1):1–2. doi: 10.1038/s41467-020-18871-1 33060586 PMC7562722

[36] Xiao SS Mi JD Mei L Liang J Feng KX Wu YB. Microbial diversity and community variation in the intestines of layer chickens. Animals (2021) 11(3):840. doi: 10.3390/ani11030840 33809729 PMC8002243

[37] Clemente JC Ursell LK Parfrey LW Knight R. The impact of the gut microbiota on human health: An integrative view. Cell (2012) 148(6):1258–70. doi: 10.1016/j.cell.2012.01.035 PMC5050011 22424233

[38] Zhou Z Nie K Huang Q Li K Sun Y Zhou R. Changes of cecal microflora in chickens following eimeria tenella challenge and regulating effect of coated sodium butyrate. Exp Parasitol (2017) 177:73–81. doi: 10.1016/j.exppara.2017.04.007 28455119

[39] Clarke G Stilling RM Kennedy PJ Stanton C Cryan JF Dinan TG. Minireview: gut microbiota: The neglected endocrine organ. Mol Endocrinol (2014) 28(8):1221–38. doi: 10.1210/me.2014-1108 PMC5414803 24892638

[40] Kim HB Isaacson RE. The pig gut microbial diversity: Understanding the pig gut microbial ecology through the next generation high throughput sequencing. Veterinary Microbiol (2015) 177(3-4):242–51. doi: 10.1016/j.vetmic.2015.03.014 25843944

[41] Dong XY Azzam MM Zou XT. Effects of dietary threonine supplementation on intestinal barrier function and gut microbiota of laying hens. Poultry Sci (2017) 96(10):3654–63. doi: 10.3382/ps/pex185 28938780

[42] Lozupone C Faust K Raes J Faith JJ Frank DN Zaneveld J. Identifying genomic and metabolic features that can underlie early successional and opportunistic lifestyles of human gut symbionts. Genome Res (2012) 22(10):1974–84. doi: 10.1101/gr.138198.112 PMC3460192 22665442

[43] Johnson DR Lee TK Park J Fenner K Helbling DE. The functional and taxonomic richness of wastewater treatment plant microbial communities are associated with each other and with ambient nitrogen and carbon availability. Environ Microbiol (2015) 17(12):4851–60. doi: 10.1111/1462-2920.12429 24552172

[44] Chen HL Zhao XY Zhao GX Huang HB Li HR Shi CW. Dissection of the cecal microbial community in chickens after eimeria tenella infection. Parasites Vectors (2020) 13(1):1–5. doi: 10.1186/s13071-020-3897-6 32046772 PMC7014781

[45] Jollife IT Cadima J. Principal component analysis: A review and recent developments. Philos Trans R Soc A: Mathematical Phys Eng Sci (2016) 374(2065):20150202. doi: 10.1098/rsta.2015.0202 PMC4792409 26953178

[46] Aleksandrzak-Piekarczyk T Puzia W Żylińska J Cieśla J Gulewicz KA Bardowski JK. Potential of lactobacillus plantarum IBB 3036 and lactobacillus salivarius IBB 3154 to persistence in chicken after in ovo delivery. Microbiol Open (2019) 8(1):e00620. doi: 10.1098/rsta.2015.0202 PMC6341040 29575743

