# Supplementary material for: Influence of Eimeria maxima coccidia infection on gut microbiome diversity and composition of the jejunum and cecum of indigenous chicken
Source: Front Immunol. 2022 Sep 5;13:994224. doi: 10.3389/fimmu.2022.994224 (PMC9483182; doi:10.3389/fimmu.2022.994224)
Supplement: Supplementary Table 1 — Data quality control (QC) statistical analysis of samples. [file Table_1.docx]

**Supplementary Table 1.**

|  |  | |  | | |  | |  | |  | |  | |  | |
| --- | --- | --- | --- | --- | --- | --- | --- | --- | --- | --- | --- | --- | --- | --- | --- |
| Sample Name | | Raw PE | | Raw Tags (#) | Clean Tags (#) | | Effective Tags (#) | | Avg Len | | GC | | Effective | |  |
|  |  | (#) | |  |  |  |  |  | (nt) | | % | | % | |  |
| J4C.1 | | 182,540 | | 169,613 | 166,669 | | 140,408 | | 423 | | 51.29 | | 76.92 | |  |
| J4C.2 | | 182,562 | | 170,853 | 167,793 | | 154,891 | | 425 | | 51.87 | | 84.84 | |  |
| J4C.3 | | 183,334 | | 170,634 | 167,410 | | 148,475 | | 427 | | 51.16 | | 80.99 | |  |
| J4I.1 | | 187,064 | | 174,636 | 171,883 | | 159,384 | | 426 | | 50.38 | | 85.2 | |  |
| J4I.2 | | 176,607 | | 163,887 | 162,368 | | 159,015 | | 406 | | 48.88 | | 90.04 | |  |
| J4I.3 | | 185,811 | | 169,918 | 166,587 | | 150,099 | | 419 | | 51.14 | | 80.78 | |  |
| J7C.1 | | 173,706 | | 163,091 | 160,306 | | 136,808 | | 425 | | 50.9 | | 78.76 | |  |
| J7C.2 | | 185,588 | | 174,916 | 172,492 | | 156,889 | | 419 | | 50.69 | | 84.54 | |  |
| J7C.3 | | 189,107 | | 176,329 | 173,144 | | 144,112 | | 425 | | 51.32 | | 76.21 | |  |
| J7I.1 | | 170,155 | | 158,046 | 155,647 | | 138,984 | | 416 | | 49.77 | | 81.68 | |  |
| J7I.2 | | 178,937 | | 157,312 | 154,142 | | 135,013 | | 416 | | 50.78 | | 75.45 | |  |
| J7I.3 | | 178,844 | | 169,786 | 168,037 | | 148,823 | | 410 | | 50.15 | | 83.21 | |  |
| C4C.1 | | 179,841 | | 171,721 | 169,814 | | 136,849 | | 413 | | 52.25 | | 76.09 | |  |
| C4C.2 | | 178,148 | | 170,224 | 168,196 | | 139,913 | | 417 | | 52.15 | | 78.54 | |  |
| C4C.3 | | 185,368 | | 177,115 | 175,180 | | 141,382 | | 418 | | 51.85 | | 76.27 | |  |
| C4I.1 | | 176,272 | | 162,537 | 160,420 | | 129,103 | | 417 | | 50.97 | | 73.24 | |  |
| C4I.2 | | 175,149 | | 161,680 | 159,685 | | 137,587 | | 416 | | 50.39 | | 78.55 | |  |
| C4I.3 | | 174,145 | | 166,705 | 164,855 | | 132,648 | | 413 | | 51.85 | | 76.17 | |  |
| C7C.1 | | 181,611 | | 165,109 | 162,363 | | 143,056 | | 419 | | 50.36 | | 78.77 | |  |
| C7C.2 | | 176,573 | | 161,396 | 158,865 | | 126,127 | | 420 | | 51.02 | | 71.43 | |  |
| C7C.3 | | 188,494 | | 172,519 | 169,784 | | 126,355 | | 419 | | 51.19 | | 67.03 | |  |
| C7I.1 | | 177,293 | | 163,378 | 161,197 | | 128,345 | | 420 | | 51.3 | | 72.39 | |  |
| C7I.2 | | 177,448 | | 165,232 | 163,021 | | 120,859 | | 418 | | 50.82 | | 68.11 | |  |
| C7I.3 | | 174,096 | | 163,991 | 161,713 | | 133,609 | | 420 | | 50.58 | | 76.74 | |  |
| Total | | 4,318,693 | | 4,020,628 | 3,961,571 | | 3,368,734 | | 10,047 | |  | |  | |  |
| Average | | 179,946 | | 167,526 | 165,065 | | 140,364 | | 419 | |  | |  | |  |

**Supplementary Table 2.**

| **Sample name** | **observed species** | **Shannon** | **Simpson** | **chao1** | **ACE** | **goods coverage** | **PD whole tree** |
| --- | --- | --- | --- | --- | --- | --- | --- |
| J4I.1 | 428 | 1.929 | 0.427 | 476.456 | 497.373 | 0.999 | 90.81 |
| J4I.2 | 477 | 1.236 | 0.253 | 510.287 | 535.817 | 0.999 | 217.306 |
| J4I.3 | 656 | 4.022 | 0.871 | 712.742 | 717.729 | 0.999 | 236.336 |
| J4C.1 | 786 | 4.14 | 0.866 | 852.04 | 852.34 | 0.999 | 159.331 |
| J4C.2 | 764 | 3.849 | 0.842 | 830.895 | 846.32 | 0.999 | 129.023 |
| J4C.3 | 406 | 2.174 | 0.504 | 441.594 | 451.39 | 0.999 | 40.167 |
| J7I.1 | 506 | 3.452 | 0.781 | 575.188 | 556.012 | 0.999 | 124.959 |
| J7I.2 | 675 | 5.282 | 0.933 | 725.968 | 718.572 | 0.999 | 177.438 |
| J7I.3 | 988 | 4.041 | 0.766 | 1049.784 | 1068.492 | 0.999 | 177.293 |
| J7C.1 | 496 | 3.708 | 0.822 | 527.5 | 532.296 | 0.999 | 40.479 |
| J7C.2 | 563 | 3.816 | 0.823 | 605.703 | 612.613 | 0.999 | 115.679 |
| J7C.3 | 556 | 3.791 | 0.814 | 573.958 | 581.982 | 1 | 117.775 |
| C4I.1 | 540 | 4.67 | 0.836 | 572.778 | 575.259 | 0.999 | 43.976 |
| C4I.2 | 546 | 4.309 | 0.763 | 588.185 | 588.305 | 0.999 | 59.811 |
| C4I.3 | 672 | 6.13 | 0.946 | 718.471 | 723.715 | 0.999 | 101.114 |
| C4C.1 | 951 | 5.946 | 0.933 | 989.319 | 1001.111 | 0.999 | 83.87 |
| C4C.2 | 1005 | 6.149 | 0.939 | 1075 | 1072.24 | 0.999 | 93.221 |
| C4C.3 | 566 | 4.977 | 0.894 | 590.93 | 600.473 | 0.999 | 44.376 |
| C7I.1 | 449 | 3.793 | 0.837 | 493.634 | 487.261 | 0.999 | 32.574 |
| C7I.2 | 574 | 4.688 | 0.841 | 804 | 676.36 | 0.999 | 59.9 |
| C7I.3 | 816 | 3.915 | 0.758 | 855.6 | 867.197 | 0.999 | 63.429 |
| C7C.1 | 443 | 3.278 | 0.682 | 458.842 | 465.188 | 1 | 51.246 |
| C7C.2 | 491 | 3.925 | 0.796 | 516.554 | 521.414 | 1 | 41.728 |
| C7C.3 | 537 | 4.264 | 0.801 | 555.679 | 557.462 | 1 | 60.656 |
