# Supplementary figures and images for: Influence of Eimeria maxima coccidia infection on gut microbiome diversity and composition of the jejunum and cecum of indigenous chicken
Source: Front Immunol. 2022 Sep 5;13:994224. doi: 10.3389/fimmu.2022.994224 (PMC9483182; doi:10.3389/fimmu.2022.994224)

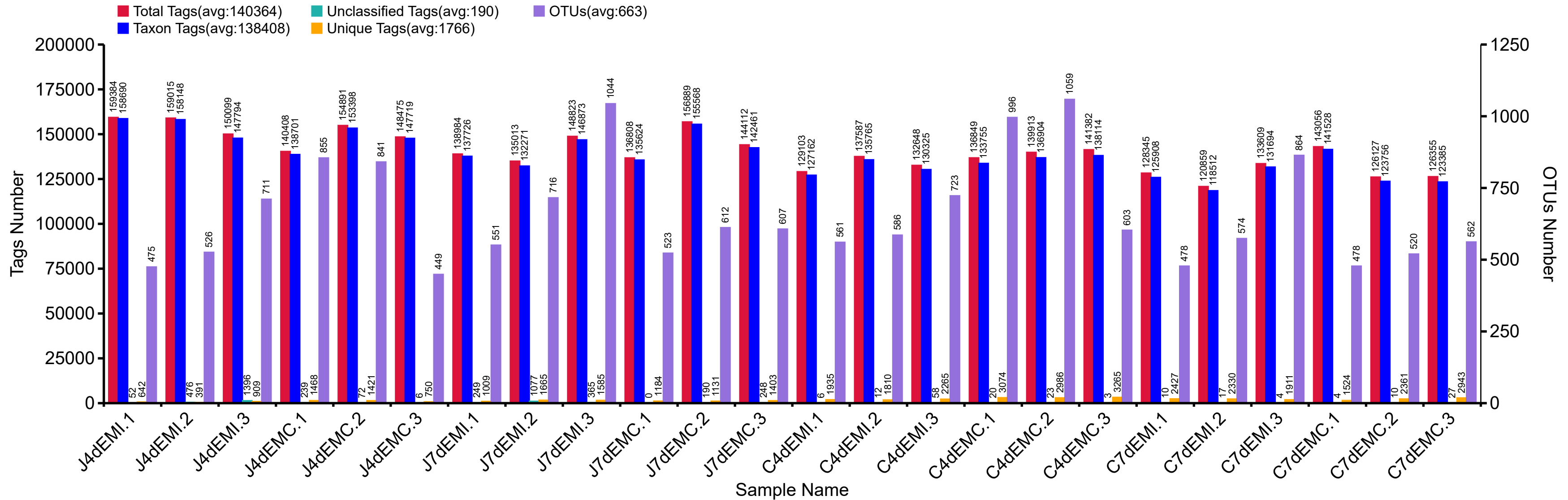

Supplement: Supplementary Figure 1 — Statistical analysis of the tags and OTUs number of each sample. [file DataSheet_1.pdf]
